# Limited-angle computed tomography with deep image and physics priors

**DOI:** 10.1038/s41598-021-97226-2

**Published:** 2021-09-06

**Authors:** Semih Barutcu, Selin Aslan, Aggelos K. Katsaggelos, Doğa Gürsoy

**Affiliations:** 1 Northwestern University, 2145 Sheridan Road, Evanston, IL 60208 USA; 2 Argonne National Laboratory, 9700 South Cass Avenue, Lemont, IL 60439 USA

**Keywords:** Computer science, Imaging techniques, Computational methods

## Abstract

Computed tomography is a well-established x-ray imaging technique to reconstruct the three-dimensional structure of objects. It has been used extensively in a variety of fields, from diagnostic imaging to materials and biological sciences. One major challenge in some applications, such as in electron or x-ray tomography systems, is that the projections cannot be gathered over all the angles due to the sample holder setup or shape of the sample. This results in an ill-posed problem called the limited angle reconstruction problem. Typical image reconstruction in this setup leads to distortion and artifacts, thereby hindering a quantitative evaluation of the results. To address this challenge, we use a generative model to effectively constrain the solution of a physics-based approach. Our approach is self-training that can iteratively learn the nonlinear mapping from partial projections to the scanned object. Because our approach combines the data likelihood and image prior terms into a single deep network, it is computationally tractable and improves performance through an end-to-end training. We also complement our approach with total-variation regularization to handle high-frequency noise in reconstructions and implement a solver based on alternating direction method of multipliers. We present numerical results for various degrees of missing angle range and noise levels, which demonstrate the effectiveness of the proposed approach.

## Introduction

**Figure 1:**
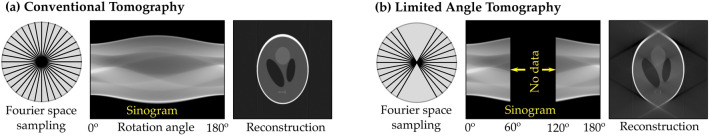
Comparison of the conventional and limited angle tomography problems.

Computed tomography (CT) has found widespread applications in various areas from diagnostic imaging to studies of materials and biological systems^1–7^. In CT, an object illuminated by x-rays is rotated while collecting projection image frames through a pixel array detector. In some CT setups, a uniform sampling of projections around the object is not possible due to the experimental setup or the extended shape of the object. For instance, in electron tomography, the angles are typically limited between − 70 to + 70$^\circ$. The challenge also appears for imaging planar objects such as integrated circuits because the light cannot penetrate the object due to increased thickness or absorption at certain angles. Sometimes, this problem is also referred to as the “missing wedge” problem since projections do not have any information about a wedge-shaped area in the Fourier space^8^; see Fig. 1 for the illustration of the problem.

There are optimization-based iterative approaches to compensate for the imaging artifacts induced by the limited projections. Algebraic reconstruction approaches, such as the algebraic reconstruction technique^9^, or the simultaneous iterative reconstruction technique^10^, often perform better than the traditional filtered-backprojection (FBP) method, at the expense of added computational cost. To better constrain the solution, compressive sensing techniques^11,12^ have been applied to promote sparsity in reconstructions. Total variation (TV) regularization is commonly used to obtain improved accuracy of the solution while suppressing high-frequency noise in reconstructions. However, a challenge is the choice of the regularization parameter, which is often performed heuristically in practical applications.

Learning-based approaches for improving the reconstructions provide an alternative. Most of the initial studies are essentially focused on image processing with pre-trained deep networks, either to in-paint sinogram images^13–16^, or to correct artifacts in reconstructed images^17,18^. Therefore, they can be considered as a nonlinear filtering operation before or after the image reconstruction step. Although successful results have been achieved using pre-trained deep networks, the effectiveness of these approaches is fundamentally limited by the training data used. While these methods offer superior results in fields like medical imaging, where training data may be readily available, they are often not applicable to the fields of microscopy due to the limited amount of datasets for training.

A better approach for applications, for which training data is not available or scarce, is to use untrained networks, called deep image priors (DIPs)^19^. With DIPs, the network architecture itself constrains the solution. Similarly to the compressive sensing approach, DIPs can perform image processing tasks like inpainting or image recovery from incomplete observations. They do so by leveraging the superior low-dimensional image representation capability of deep generative networks. Recently, DIPs are proposed for the CT problem as a post-processing technique after tomographic reconstruction^20^. In a related study, DIP is used for the limited angle problem but for diffraction tomography^21^, where the measured images are in the Fourier space. In parallel, Yang et al.^22^ introduced a similar self-training optimization approach by combining DIP and tomographic reconstruction through a single network. While they showed great improvements compared to non-learning approaches, they also reported the instability of the method when measurements are noisy.

In this paper, we apply DIP to the limited angle problem for tomography, and extend DIP by incorporating regularization to improve robustness to noise. To effectively solve the problem, we also introduce an augmented Lagrangian type approach, which enables the use of the alternating direction method of multipliers (ADMM). Because with ADMM, we can break the imaging problem into smaller and well-defined sub-problems that can be solved independently, we can effectively map those sub-problems onto available computing resources. To further improve the method, we extend the work of Yang et al.^22^ by incorporating a fully-connected network before the DIP network, as the reconstruction is heavily dependent on the inverse projection operator, and a fully connected network can be trained as one. Through numerical experiments, we analyze the convergence behavior of the method and show that our approach can outperform standard methods like FBP and TV, based on the structural similarity index (SSIM) and peak signal-to-noise ratio (PSNR).

## Methods

In this section, we present our approach to model the inverse problem of limited angle tomography and our solution approach. Our approach uses a generative model for constraining the solution, an approach similar to deep image priors (DIP)^19^. We also combine this approach with regularization methods for improved robustness of the reconstruction in noisy settings.

### The inverse problem

The limited angle tomography acquisition process is well approximated by a linear imaging model as:

1 $$\begin{aligned} d = Rx + n \end{aligned}$$

where $R:{\mathbb {R}}^{Mx1}\rightarrow {\mathbb {R}}^{Nx1}$ is the x-ray projection operator that defines a mapping from a three-dimensional object represented in a vector form, $x\in {\mathbb {R}}^{Mx1}$, to a set of projection data, $d\in {\mathbb {R}}^{Nx1}$, with measurement noise, $n\in {\mathbb {R}}^{Nx1}$. A common approach to solve the inverse limited angle tomography problem, is to carry out the optimization:

2 $$\begin{aligned} {\hat{x}} = {{\,{\mathrm{arg\,min}}\,}}_{x} \Vert Rx - d\Vert _1 + \alpha TV\left( x\right) \end{aligned}$$

*TV* is the total variation distance, and is defined as $TV\left( x\right) = \Vert \nabla x \Vert _1$. The first term is the data fidelity term measured by the $\ell _1$ norm and the second term is the regularization term to favor piecewise-constant solutions. $\lambda$ is the regularization parameter that controls the trade-off between these terms. $\ell _1$ norm is chosen for data fidelity term due to the increased reconstruction quality compared to $\ell _2$ norm at early experimentation.

To leverage deep generative models for effectively constraining the solution, we will describe *x* with, $G_w:{\mathbb {R}}^{Nx1}\rightarrow {\mathbb {R}}^{Mx1}$, where *w* represents the weights of the deep neural network. This leads to the optimization problem of the form:

3 $$\begin{aligned} {\hat{w}} = {{\,{\mathrm{arg\,min}}\,}}_{w} \Vert R G_w (d) - d \Vert _1 + \alpha \Vert \nabla G_w (d) \Vert _1. \end{aligned}$$

Because the network defines a mapping from the data domain to the object domain, it can be seen as an approximation to the inverse x-ray projection operation. Therefore, it captures both attributes of physics-based inversion and deep image prior into a single deep neural network.

**Figure 2:**
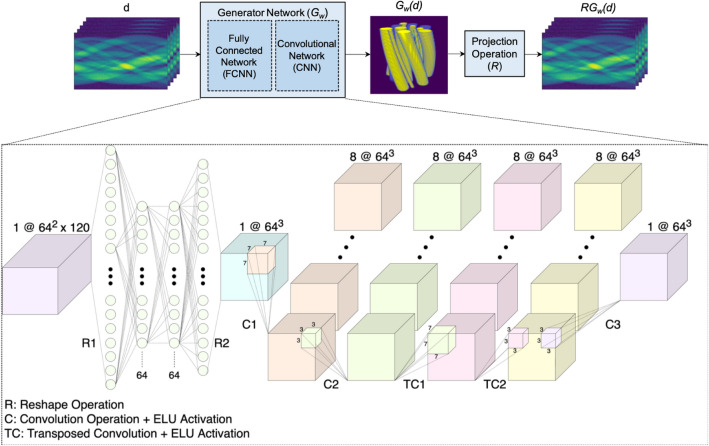
Illustration of the operators and deep neural network architecture^23^.

### Solution of the inverse problem

For solving Eq. (3), we first convert the problem into a constrained optimization problem by using an auxiliary variable, and use the alternating direction method of multipliers (ADMM) for the solution^24^. The constrained problem formulation is given as:

4 $$\begin{aligned} {\hat{w}}, {\hat{y}} = {{\,{\mathrm{arg\,min}}\,}}_{w,y} \Vert R G_w (d) - d \Vert _1 + \alpha \Vert y \Vert _1 \ \ \ {\text {subject to}} \ \ \ \nabla G_w (d) = y \end{aligned}$$

and the corresponding augmented Lagrangian is written as follows,

5 $$\begin{aligned} L(w, y, z) = \Vert R G_w (d) - d \Vert _1 + \alpha \Vert y \Vert _1 + z^T\left( \nabla G_w (d) - y\right) + \tau /2\Vert \nabla G_w (d) - y\Vert _2^2, \end{aligned}$$

where $\tau > 0$ is an auxiliary parameter and $z\in {\mathbb {R}}^{Nx1}$ is introduced as the dual variable. We use ADMM to minimize Eq. (5) by iteratively solving for *w* and *y*, followed by the update of the dual variable *z*, that is,

6 $$\begin{aligned} w^{k+1}&= {{\mathrm{arg\,min}}}_{w} \left\{ \Vert R G_w (d) - d \Vert _1 + \tau /2 \Vert \nabla G_w (d) - y^k+z^k/\tau \Vert _2^2\right\} \end{aligned}$$

7 $$\begin{aligned} y^{k+1}&= {{\mathrm{arg\,min}}}_{y} \left\{ \alpha \Vert y \Vert _1+ \tau /2 \Vert \nabla G_{w^{k+1}}(d) - y +z^k/\tau \Vert _2^2\right\} \end{aligned}$$

8 $$\begin{aligned} z^{k+1}&= z^k + \tau \left( \nabla G_{w^{k+1}}(d) - y^{k+1} \right) . \end{aligned}$$

At each ADMM iteration, *L*(*w*, *y*, *z*) is minimized over each variable by fixing the other variables until convergence.

The operators for the problem and the network architecture architecture are illustrated in Fig. 2. Because the deep generative network in sub-problem Eq. (6) is used to both represent the inverse x-ray projection operation and the deep image prior, we use two cascaded neural network architectures: a fully connected network followed by a convolutional network. In this configuration, the input projections are mapped to the reconstruction domain by the fully connected layers, and then they are converted to a three-dimensional array. Convolutional layers then convert the features of the 3D tensors to minimize sub-problem Eq. (6).

The proposed solution utilizes the deep image priors in order to improve the reconstruction. The networks implicitly optimize the weight for inpainting missing parts of the projections with the help of the physical forward model. Although there is missing information, low-level statistical distribution learning is used to estimate the 3D object structure, and the network reconstructs the 3D object by filling out the missing information in the measurements. The capability of the network to inpaint the missing parts of the projections depends on the convergence of the self-learning process to optimize the weights of the network.

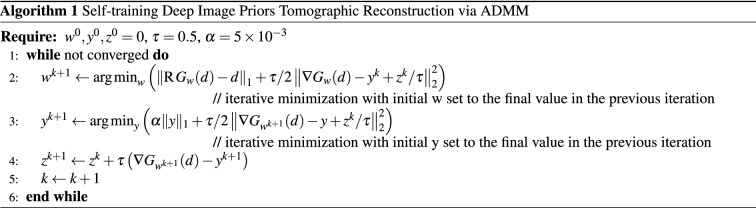



### Implementation details

Cascaded architecture of the generative network is shown in Fig. 2 for an input of 120 projections acquired from an object with a support of $64 \times 64\times 64$ pixels. First part of the network consists of four fully-connected layers where hidden layers have 64 nodes each. Each fully-connected layer is followed by a hyperbolic tangent activation function, a layer normalization operation, and a dropout layer with a dropout ratio of 0.25. Then, the output of the fully-connected network is reshaped into $64\times 64\times 64$. The following convolutional network consists of two 3D convolutional layers, two 3D transposed convolutional layers, and one 3D convolutional layer with kernel sizes of 7, 3, 7, 3, and 3, respectively. Padding is applied for maintaining the size and stride size is kept at 1, while 8 filters are used for each convolutional layer. Similarly, each convolutional layer is followed by a Exponential Linear Unit (ELU) activation and a layer normalization operation, and the output of the last convolutional layer is the reconstruction of 3D object.

Algorithm 1 presents the pseudo-code of our ADMM algorithm. For the minimization of sub-problem Eq. (6), we use the Adam optimizer^25^ with a learning rate of $2\times 10^{-5}$, and the two momentum terms are equal to 0.5 and 0.999. We experimented with different values for the total variation regularizer parameter $\alpha$ empirically, and we found that values between $1e-3$ and $1e-5$ give reasonable results for different objects and different levels of desired piece-wise smoothness. This change depends on the object, and the reconstruction is not too sensitive to change of the value of $\alpha$. We initialize *w*, *y*, and *z* with zeros at the first iteration, and each of the inner optimizations for w [Eq. (6)] and y [Eq. (7)] are initialized with their corresponding values from their converging values at the previous outer iterations. *tau* is initialized with the value of 0.5, and it is updated based on Eqs. (9), (10) and (11 )at every outer iteration for optimizing the value for the best convergence behavior. The number of iterations for estimating the network weights is set to 1000 for each ADMM iteration. Similarly to other DIP implementations, we use early stopping as a regularization to avoid over-fitting the noise.

9 $$\begin{aligned} \epsilon _1^{k}&= \Vert \nabla G_{w^{k}} (d) - y^{k} \Vert _2 \end{aligned}$$

10 $$\begin{aligned} \epsilon _2^{k}&= \tau ^{k} \Vert \nabla G_{w^{k}} (d) - \nabla G_{w^{k-1}} (d) \Vert _2 \end{aligned}$$

11 $$\begin{aligned} \tau ^{k+1}&= {\left\{ \begin{array}{ll} 2\tau ^{k}, & {\text {if }} \epsilon _1\ge 10\epsilon _2\\ \frac{1}{2}\tau ^{k}, & {\text {if }} \epsilon _2\ge 10\epsilon _1 \end{array}\right. } \end{aligned}$$

We use soft-thresholding^26^ for explicitly solving the subproblem Eq. (7). For simplicity, we assume element-wise operations for taking the absolute value, comparing to zero, and dividing. the resulting update for *y* is:

12 $$\begin{aligned} y^{k+1} = \frac{\nabla G_{w^{k+1}}(d) + z^{k}/\tau }{\left| \nabla G_{w^{k+1}}(d) + z^{k}/\tau \right| }\max \left\{ 0, \left| \nabla G_{w^{k+1}}(d) + z^{k}/\tau \right| - \alpha /\tau \right\} . \end{aligned}$$

We normalize the range of input variables to avoid overflowing of gradients. The normalization is done in two steps: first, z-score normalization is done to maintain a zero mean and unit variance; and second, min-max normalization is done to obtain values between 0 and 1. We apply normalization both to the input and the computed projections. In other words, we normalize the x-ray transform operator, R. Besides, we apply a layer normalization^27^ to every layer of the network to improve the convergence and accuracy of the network.

**Figure 3:**
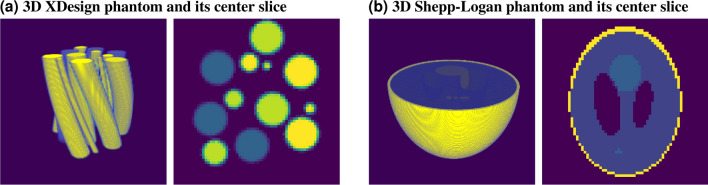
Simulated 3D objects^23,28^ and their center slices.

## Results

### Simulation setup

To evaluate the effectiveness of the proposed method against the state-of-the-art methods, numerical tests are performed on two simulated phantoms with support of $64 \times 64 \times 64$ pixels. One phantom is a simulated 3D object consisting of two spiral-shaped materials created by XDesign^29^, that is made of a strongly absorbing material, gold (Au), and a weakly absorbing material, zinc oxide (ZnO). The densities of these materials are 19.32 and 5.606 g/cm$^3$, respectively. A 5 keV energy beam is used to simulate the refractive index values. The XDesign Phantom and its center slice are illustrated in Fig. 3a. The second phantom is chosen to be the 3D Shepp–Logan phantom from TomoPy^30^; see Fig. 3b for the illustration of the bottom half of the 3D object and its center slice. The objects are chosen to be sparse in 3D space to observe the artifact removal in limited angle tomography reconstructions more clearly.

For simulations throughout the paper, projections are acquired via a limited angle tomography setup where the objects are rotated around their common axes. Projections are simulated by $1^{\circ }$ separation in the limited ranges of $0^{\circ }$–$120^{\circ }$ and $0^{\circ }$–$150^{\circ }$; corresponding to $60^{\circ }$ and $30^{\circ }$ of missing wedge information, respectively. In addition, Gaussian noise with increasing variance is added to test the noise removal capabilities of the proposed method, resulting in projections that have noise with variances 0.5, 2.5, 5, and 10. We assumed Gaussian noise on measurements because we mainly target transmission-type microscopes, where the photon fluence on the sample is reasonably high. However, for microscopes that use secondary signals like fluorescence emission or gamma radiation, a Poisson data formation model may be a better choice.

**Figure 4:**
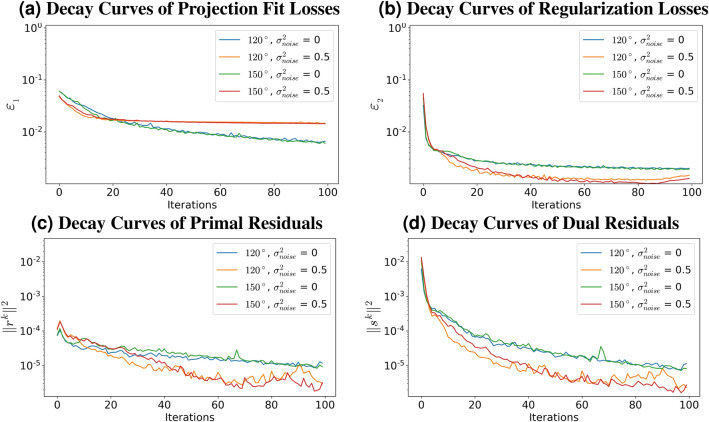
Convergence curve plots.

### Analysis of convergence behavior

In this section, we discuss the convergence behavior of the proposed algorithm by evaluating how different losses behave as a function of each iteration. In Fig. 4a, we show the term $\Vert R G_{w^k} (d) - d \Vert _1$ as a function of iteration k. It is a measure of how well the generated projections based on the reconstructed 3D object by the deep neural network fit the available data, for different number of projections and amounts of noise.

13 $$\begin{aligned} \epsilon _1 = \Vert R G_{w^k} (d) - d \Vert _1 \end{aligned}$$

The amount of noise in the projections is the main factor determining the convergence of the projection fit. Although all setups are able to minimize this loss with increasing number of iterations, the error decreases to a significantly lower value in noise-free cases because it is not possible for the generated projections to fit Gaussian noise. This behavior is not only expected but also desired since the targeted reconstructions are required to be free of noise. Similarly, the error minimization of projection fit for noisy setups is adjusted by the regularization loss. Unlike the effect of noise, the effect of the missing wedge information is minimal on the convergence. With increased number of projection angles, the decay of the error term is slightly improved.

The second loss function to update the deep network is the loss derived from the regularization term. The auxiliary ADMM variable, *y*, which should converge to the gradient of a noise free reconstruction, is updated using total variation (TV) regularization to have minimum values except at the edges. Then, the network weights are updated by minimizing:

14 $$\begin{aligned} \epsilon _2 = \tau /2 \Vert \nabla G_w^k (d) - y^k+z^k /\tau \Vert _2^2 \end{aligned}$$

As shown in Fig. 4b, unlike the convergence of the projection fit loss, change in the error amount of the regularization loss has a positive correlation with the variance of the noise in the projections. Since the target is for the reconstruction to have piece-wise smoothness, reconstructions with high variance are effected by regularization more, and the error is minimized accordingly for noisy projections. A trade-off between the effects of regularization and the projection fit is apparent in noisy projection setups, where increased piece-wise smoothness means worse fit to the sinograms. Due to this trade-off, decay curves show an increase in error for the regularization term with the increased number of iterations while the generated projections converge to the noisy projections. Similarly to the projection fit decay curve, the error for regularization also achieves smaller values for increased number of projections with decreasing effects of missing wedge artifacts.

ADMM convergence can be analyzed by the observation of the decay curves of primal and dual residuals of the ADMM framework. The primal and dual residuals, $r^{k+1}$ and $s^{k+1}$, respectively, can be derived from Eq. (5) as follows, where k is the outer iteration number:

15 $$\begin{aligned} r^{k+1}= & {} y^{k+1} - \nabla G_{w^{k+1}} (d) \end{aligned}$$

16 $$\begin{aligned} s^{k+1}= & {} \tau \left\{ \nabla G_{w^{k+1}} (d) - \nabla G_{w^{k}} (d)\right) \} \end{aligned}$$

The derived primal and dual residuals demonstrate the behavior of the update steps taken in the ADMM iterations. While the primal residual is a measure of how well the reconstructions converge towards the auxiliary variable *z*, the dual residual shows how much the reconstruction is updated towards the solution. While Figs. 4c and d demonstrates that residuals are minimized with increased number of iterations for each setup, the amount of decrease is more for the noisy reconstructions due to the increased divergence from the initial reconstructions and higher number of degrees of freedom they have.

### Reconstructions and comparisons

In this section, we show the reconstructions for different tomographic angle ranges, list the methods in the literature to be compared to the proposed approach, present the reconstructions for the simulations for all methods, and discuss the results qualitatively and quantitatively.

**Figure 5:**
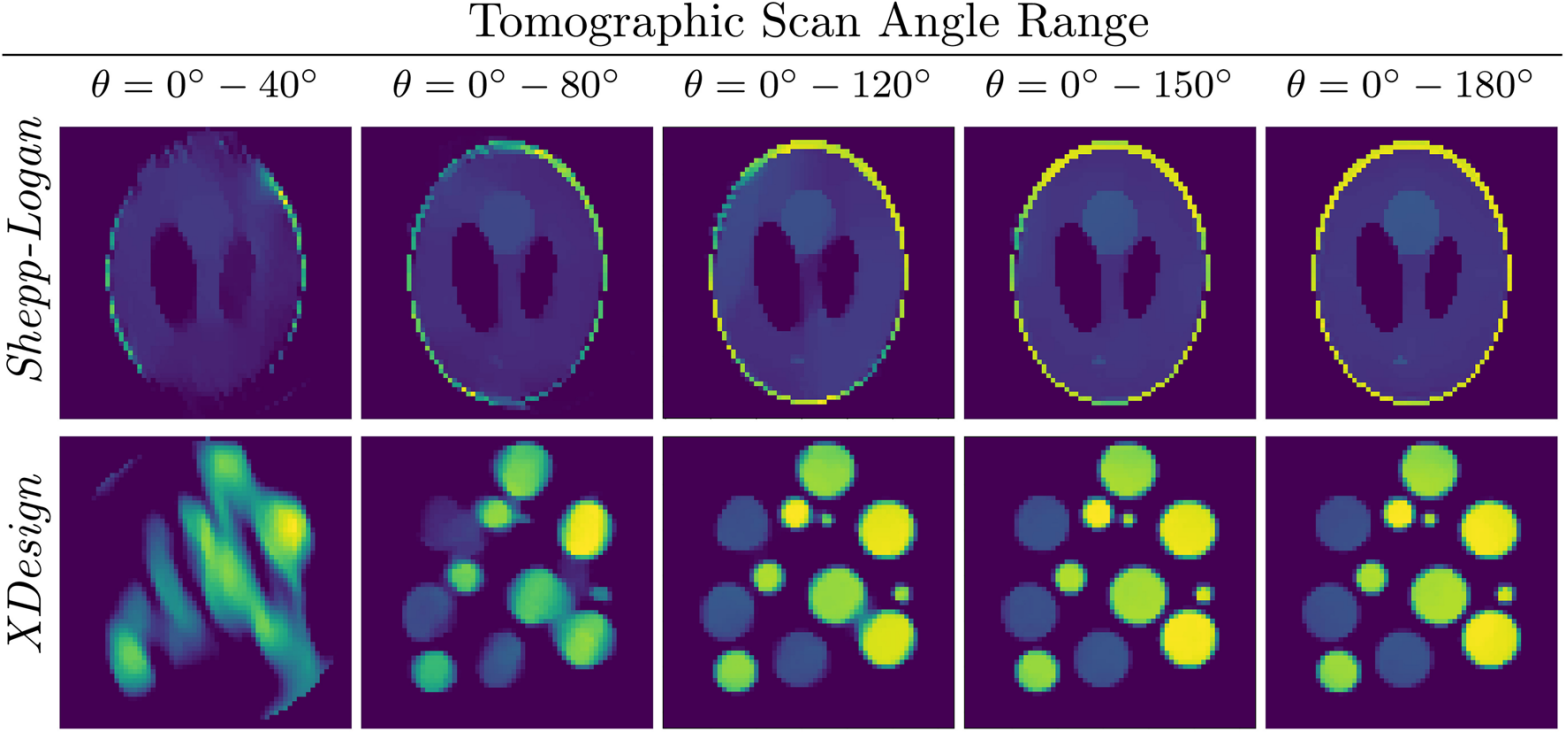
Reconstructions for projection in varying angles from noise-free projections.

In Fig. 5, the center slices of 3D reconstructions of the two simulated objects with different tomographic scan angles in the noise-free case are demonstrated. As can be seen, reconstruction for $0^\circ$–$150^\circ$ case is the reconstruction is almost identical with the full scan angle case, and the degradation in the reconstruction for $0^\circ$–$120^\circ$ case is minimal with some small visible artifacts. With the increased missing wedge size, the reconstruction quality decreases as expected. However, the reconstruction for the Shepp–Logan phantom, which is a more compact object, is quite reasonable even for even the $0^\circ$–$40^\circ$ case, where the reconstruction for the $0^\circ$–$80^\circ$ does not even show easily visible artifacts. In contrast, the limitation of the approach for a more complex object such as the XDesign phantom can be observed for small tomographic angle range reconstructions. While the reconstruction for the $0^\circ$–$80^\circ$ case is acceptable, the structure of the object is lost in the $0^\circ$–$40^\circ$ case. In addition to the reconstructions in Fig. 5, reconstructions of a slab-shaped 2D object with square inclusions are provided in Supplementary Fig. S2 to show the effectiveness of the algorithm for different types of objects. It is observed that the proposed method also produces successful reconstructions with a slab-shaped object.

#### Comparative studies

To evaluate the improvements brought by the proposed method over the ones in the literature, reconstructions from the proposed algorithm are compared to the reconstructions from state-of-the-art algorithms. The baseline algorithm used for comparison is called GridRec^31,32^, a simple filtered-back projection method^33^ implemented by the application of interpolation in Cartesian coordinates instead of polar coordinates. GridRec is demonstrated as the primal reconstruction method that does not take any steps to compensate for missing wedge artifacts or noise. A more elaborate and advanced algorithm is developed by Yang et al.^22^, deploying a self-training approach with a Generative Adversarial Network (GAN), called GANrec, and aiming at accurate slice-by-slice reconstructions by the use of a generative network with the help of a discriminator network loss. Although the method is successful for reconstructions in noise-free cases and eliminates some of the missing wedge artifacts, it is not robust to noisy projection inputs.

To understand the effectiveness of the noise removal by regularization in the proposed approach, a Total Variation (TV) reconstruction algorithm^34^ is used, which deploys a total variation regularization integrated into back-projection algorithm to lower the noise and missing wedge artifacts. For TV, an efficient implementation called TIGRE^35^ is used for reconstructions. The Deep Image Prior (DIP) algorithm is also tested by designing a DIP network as shown in Supplementary Fig. S1. The convolutional network implemented in DIP is chosen to be deeper than the cascaded network in the proposed model, and has the same number of trainable parameters. The network is trained in the same ADMM framework with the addition of total variation regularization. For GridRec, GANrec, TV, DIP, and the proposed method, the same forward projector^36^ is used, and GANrec is implemented in PyTorch for a fair comparison.

**Figure 6:**
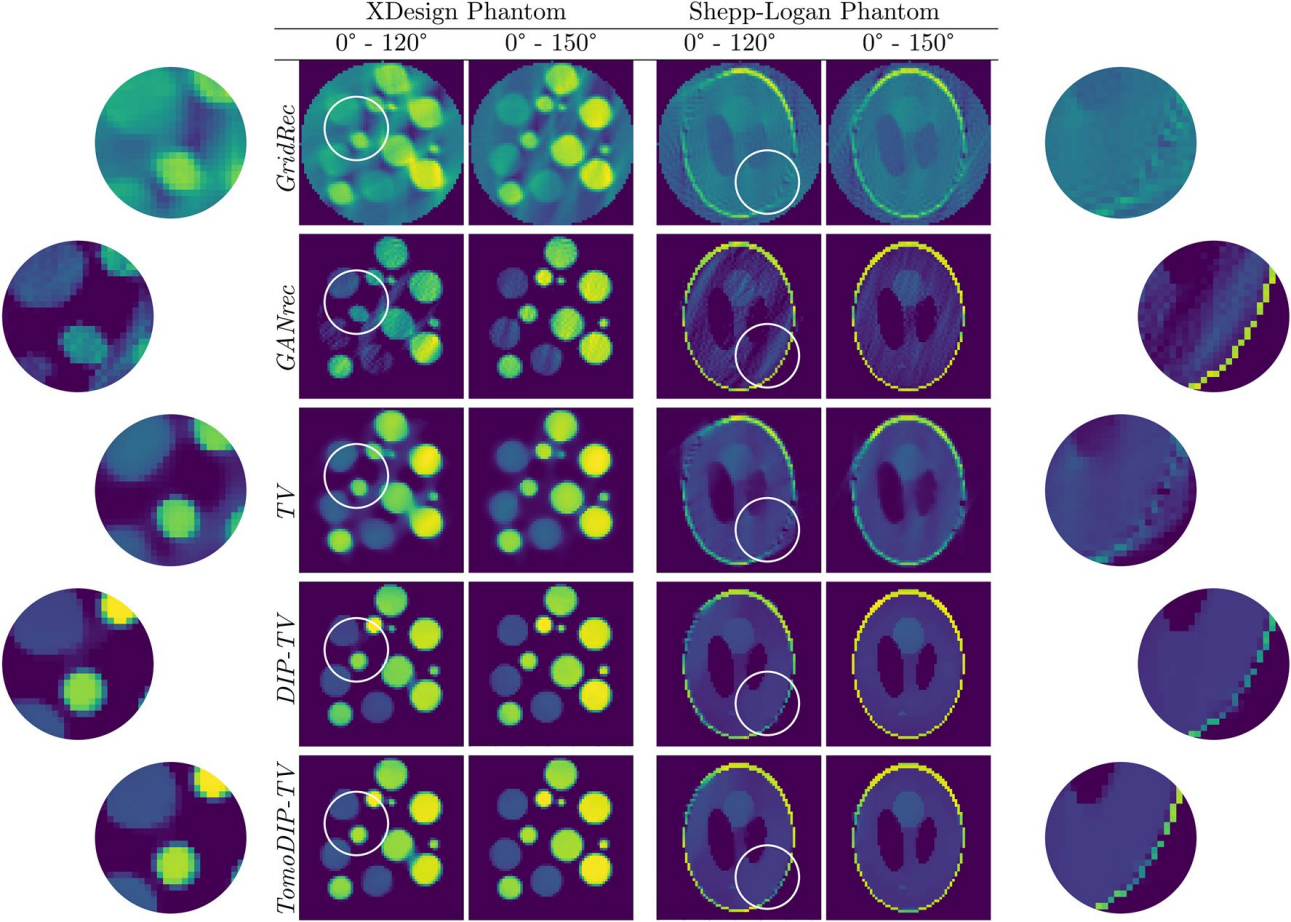
Reconstruction comparison for the XDesign phantom and the Shepp–Logan phantom for the noise-free case.

#### Comparisons

In Fig. 6, center slices of the 3D reconstructions of GridRec, GANrec, TV, Deep Image Priors with TV (DIP-TV), and the proposed method (TomoDIP-TV) from noise-free projections are shown. For both angle ranges and phantoms, GridRec fails to generate comparable results due to the missing projections. Reconstructions from GANrec are significantly improved compared to the GridRec. GANrec is able to eliminate elongation artifacts at the edges of the sections of the object, although it shows some streaking artifacts in the areas that should have been piece-wise constant. Details of the improvements at the edges are more visible in the zoomed images; however, streaking artifacts are shown to be above the desired limits for the low number of projections. This effect is more observable for the XDesign phantom. TV method improves the piece-wise smoothness with the help of regularization, but fails at compensating for missing wedge artifacts. As shown in the zoomed images, although the streaking artifacts are reduced, borders of the objects experience notable artifacts. Besides, even though the reconstructions for fewer missing wedge data (0$^\circ$– 150$^\circ$) are close to the ground truth, effects of over-regularization can be seen at the edges. However, DIP-TV greatly reduces the artifacts and reconstructs the objects more accurately. The proposed method improves the reconstruction quality even more by reducing the artifacts at the edges to very low levels. This effect can be observed in detail in the zoomed images.

In Table 1, Structural Similarity Index Measure (SSIM) and Peak Signal-to-Noise Ratio (PSNR) for the noise-free reconstructions are shown. The quantitative measures of the reconstructions mostly align with observations in the qualitative analysis, and the proposed method performs the best overall. On the other hand, in particular setups, DIP-TV method generates slightly higher measures when there is enough data and less variance in the object values. While GridRec has the worst measures, GANrec and TV show their advantages in different setups.

**Table 1:** Quantitative comparison of reconstruction qualities from noise-free projections (Best SSIM and PSNR results per number of projections are shown in bold).

3D Noise free reconstruction	XDesign phantom				Shepp–Logan phantom			
	0$^\circ$- 120$^\circ$		0$^\circ$- 150$^\circ$		0$^\circ$- 120$^\circ$		0$^\circ$ - 150$^\circ$	
	ssim	psnr	ssim	psnr	ssim	psnr	ssim	psnr
GridRec	0.49	60.90	0.56	62.50	0.39	62.51	0.42	63.20
GANrec	0.74	65.76	0.90	71.23	0.79	69.43	0.86	69.06
TV	0.86	70.62	0.93	74.44	0.80	68.43	0.88	70.41
DIP-TV	0.90	72.37	0.91	72.48	0.83	68.55	**0.97**	**76.18**
TomoDIP-TV	**0.91**	**73.15**	**0.94**	**76.01**	**0.86**	**69.41**	0.95	73.98

**Figure 7:**
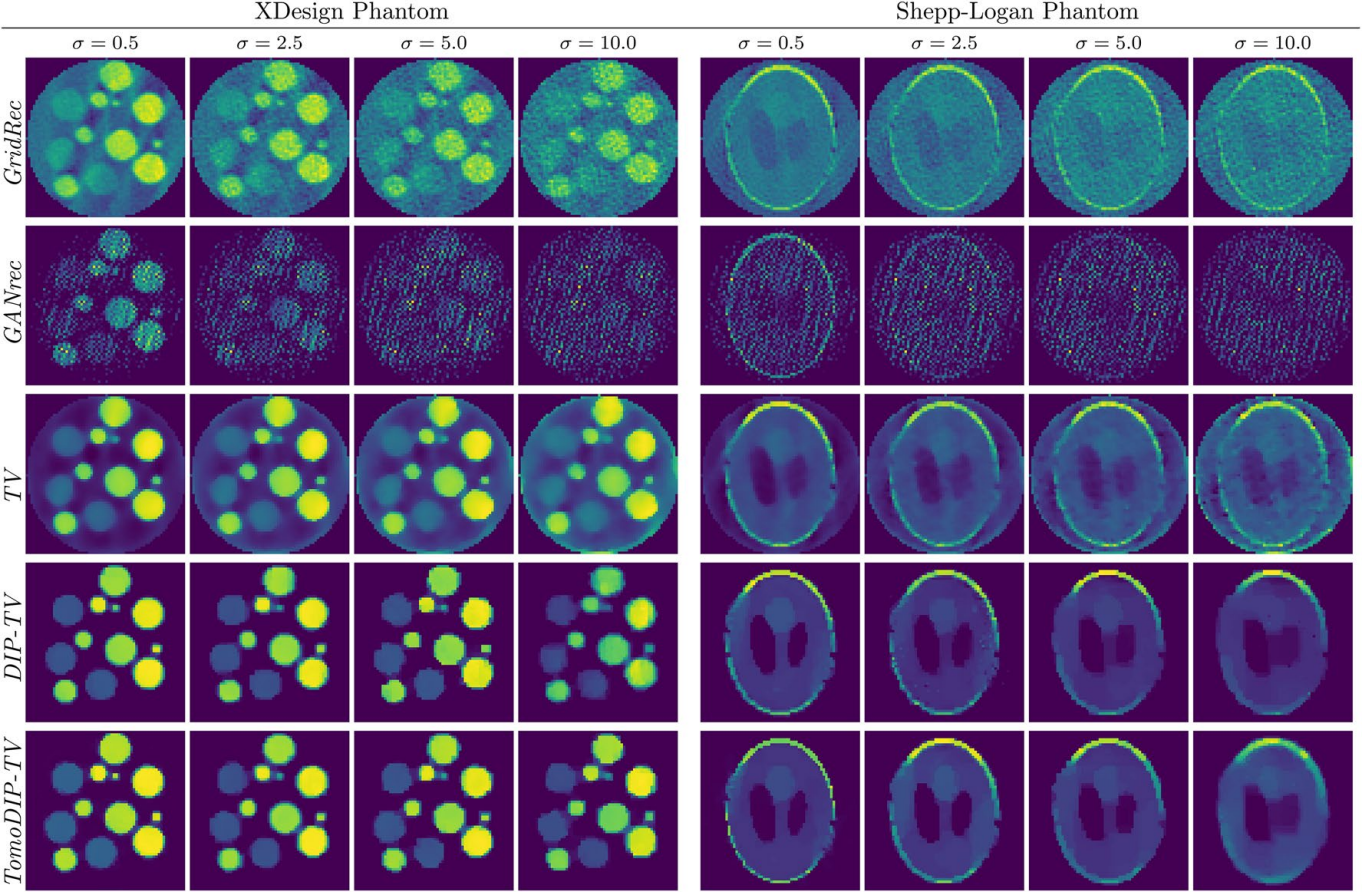
Reconstruction comparison for the XDesign phantom and the Shepp–Logan phantom for different noise levels and for projections between 0$^\circ$ and 150$^\circ$.

The effect of increased noise variance in the projections is evaluated in Fig. 7 where center slices of the 3D reconstructions using GridRec, GANrec, TV, DIP-TV, and TomoDIP-TV from noisy projections are shown. For the simulations, the variance of the Gaussian noise is varied between 0.5 and 10, while the projection angle range is kept the same ($0^\circ$–$150^\circ$). As expected, GridRec and GANrec do not perform well when the input projections are noisy. In fact, GANrec suffers more from the increased noise since there is no mechanism in the algorithm to prevent the generated projections from fitting the noisy sinograms, creating even noisier reconstructions. TV performs significantly better than the previous two methods, but it suffers from poor reconstruction quality at the edges. Moreover, the reconstruction quality is greatly reduced with increased noise, where the trade-off between missing wedge artifacts and regularization is more apparent. DIP-TV takes advantage of the regularization effect of the deep image priors and reconstructs the 3D object well, even for the setups with high noise variance. However, it fails to eliminate artifacts to recover the object structure when the noise variance is increased beyond its limits. On the other hand, TomoDIP-TV takes advantage of the fully connected network to reconstruct the object structure and improves the quality with the help of DIP and TV regularization. Although the reconstructions for projections with high noise variance are far from ideal, the object structure is preserved more compared to the previous methods.

Quantitative analysis of the 3D reconstructions from noisy projections can be seen in Table 2. Most of the SSIM and PSNR values are consistent with the qualitative observations and highly favor the proposed method as the best reconstruction method. While reconstructions using GANrec have the lowest SSIM and PSNR values, the increase towards the proposed method in SSIM is notably higher than the increase in PSNR. In some cases, the PSNR values are slightly lower for the proposed method, whereas the SSIM values are significantly higher. For those setups, qualitative evaluations of the reconstructions in Fig. 7 show that even though the PSNR is lower, the structural integrity of the reconstructions is higher for the proposed method than for the rest of the algorithms.

**Table 2:** Quantitative comparison of reconstruction qualities from noisy projections between 0$^\circ$ and 150$^\circ$ (Best SSIM and PSNR results per noise level are shown in bold).

3D noisy reconstruction	XDesign phantom								Shepp–Logan phantom							
	$\sigma ^2 = 0.5$		$\sigma ^2 = 2.5$		$\sigma ^2 = 5$		$\sigma ^2 = 10$		$\sigma ^2 = 0.5$		$\sigma ^2 = 2.5$		$\sigma ^2 = 5$		$\sigma ^2 = 10$	
	ssim	psnr	ssim	psnr	ssim	psnr	ssim	psnr	ssim	psnr	ssim	psnr	ssim	psnr	ssim	psnr
GridRec	0.55	62.30	0.52	61.98	0.49	61.62	0.46	61.06	0.39	62.83	0.34	62.34	0.31	61.43	0.29	61.12
GANrec	0.49	61.89	0.30	59.56	0.25	58.90	0.24	58.75	0.38	64.25	0.26	62.55	0.24	62.22	0.22	61.98
TV	0.79	71.97	0.73	69.06	0.69	67.48	0.63	65.38	0.64	68.90	0.54	67.49	0.50	66.77	0.45	65.60
DIP-TV	0.89	69.01	0.84	67.52	0.83	66.70	0.73	**71.11**	0.85	69.02	0.79	67.80	0.75	66.87	**0.71**	66.39
TomoDIP-TV	**0.97**	**75.11**	**0.92**	**72.91**	**0.90**	**70.48**	**0.81**	66.82	**0.87**	**69.81**	**0.81**	**68.10**	**0.77**	**67.34**	**0.71**	**66.42**

**Figure 8:**
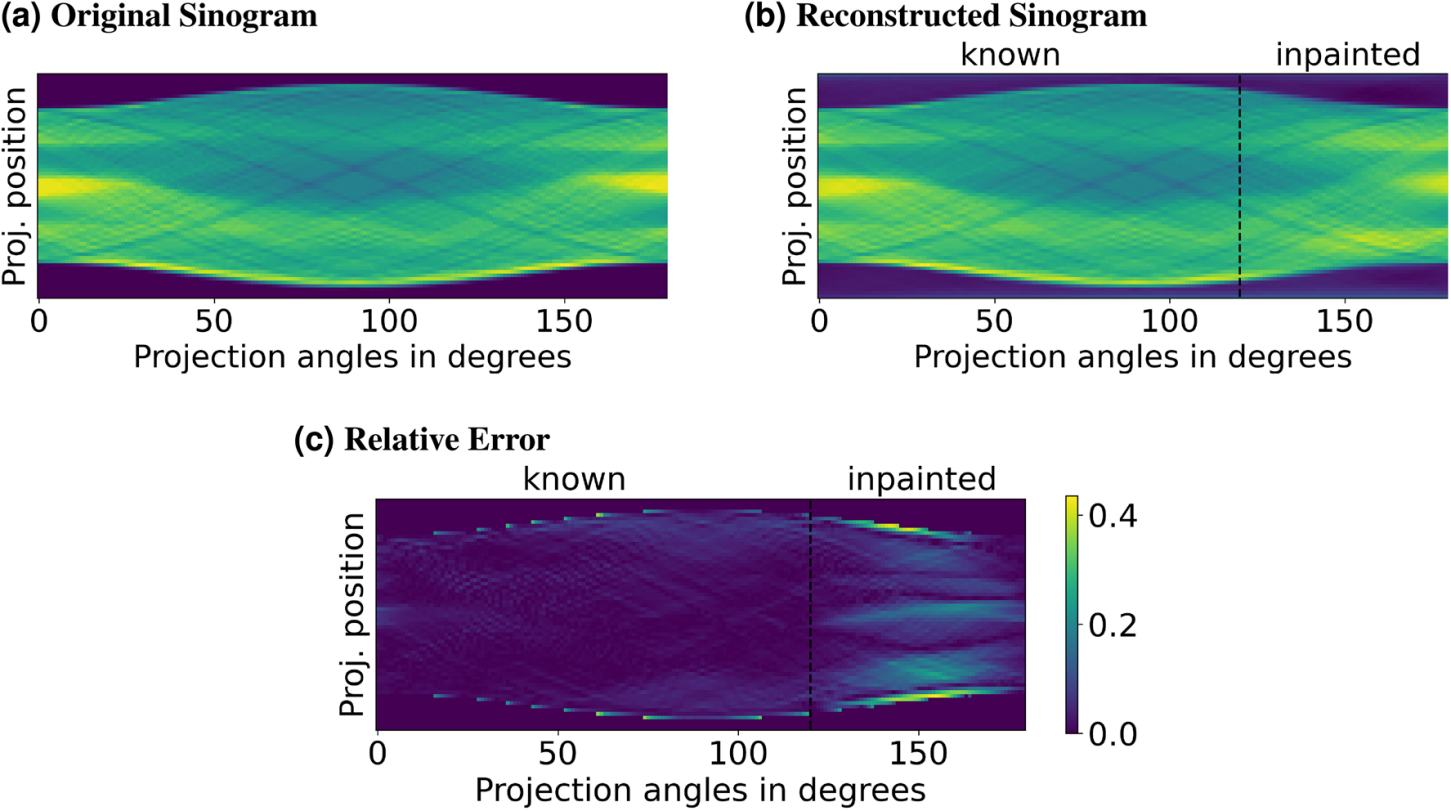
Reconstructed sinogram for noise-free $0^\circ - 120^\circ$ projections, the sinogram from the original object for Shepp–Logan phantom, and the relative error between them.

Although the sinogram completion effect of the DIP method is not explicit, it can be observed in sinograms of the reconstructed objects. A comparison of the complete sinogram for the Shepp–Logan phantom and the reconstructed sinogram for the noise-free case is given in Fig. 8. As observed, the missing part of the sinogram is constructed using only the information from the other parts of the sinogram, which do not necessarily give straightforward information about the missing part. This difference shows the advantage of the DIP method over the techniques in the literature, which lack the ability to complete the sinogram information in detail.

## Discussion

In computed tomography, uniform sampling around the object might not always be possible, creating the so-called missing-wedge problem. Reconstructions using the traditional methods such as filtered back-projection show elongation, streaking, and ghost tail artifacts. Regularizers can be employed to reduce the effects of these artifacts. An example is the TV method which incorporates total variation regularization into the back-projection operation based on preliminary information or assumptions on the object. Although this approach is successful at increasing the reconstruction quality, artifacts cannot be eliminated completely; see Fig. 6. On the other hand, deep image priors (DIP) architecture demonstrates that the whole process could be learned by self-training the network. The convolutional network employed becomes an “optimized regularizer” to reduce the artifacts. Using this idea, we have implemented a DIP network to reconstruct the 3D object directly from the projections. To incorporate total variation regularization, we applied self-training in an ADMM framework, which is able to decompose the problem into more manageable sub-problems. The resulting reconstructions showed that DIP-TV approach increased the reconstruction quality notably, eliminating both missing wedge and noise artifacts (see Figs. 6 and 7).

Although we have shown that the DIP-TV method improves the reconstruction of 3D objects compared to the methods in the literature, the idea of the DIP is to regularize an existing solution. In DIP computed tomography reconstructions, the convolutional network is responsible for the inverse projection operation in addition to being a regularizer. However, inverse projection is a mapping operation, and it is shown that it can be modeled with a fully connected network more accurately. Thus, we have designed a cascaded network consisting of fully connected and convolutional layers as shown in Fig. 2, and replaced the fully convolutional network with it. Although having a smaller regularizer network, Figs. 6, 7, and Tables 1, 2 show that there is a considerable improvement in the reconstructions with the proposed method. With a more accurate modeling of the inverse operation, it can be seen that edges are more well-defined, and variation of reconstructed values is less for the proposed approach.

Despite the improved reconstruction quality, reconstruction time is a significant drawback for the proposed method. The method is a self-training algorithm, and it requires the network to be trained for each unique reconstruction, making the proposed method considerably slower compared to the supervised learning methods or the iterative reconstruction methods. For the simulations, the training is done on Nvidia GeForce RTX 2080 Super graphics card, where each inner ADMM iteration took approximately 0.37 s, resulting in 431 min to reconstruct an object on average. The reconstruction time increases linearly with the increased number of voxels to be reconstructed, meaning an exponential increase for increased size in one dimension. This requires the size of the object to be limited for reasonable reconstruction times. However, experimental trials on using transfer learning applications showed that there is a possibility of reconstructing similar objects with the trained networks by minimal additional training, i.e., transfer learning, which can lower the reconstruction time up to 20–100 times. Starting the self-training with the optimized weights of the network from a previous reconstruction leads to a need for a smaller change in the network weights, which reduces the reconstruction time significantly. The temporal evaluation of the weights of the network in a previous reconstruction would mean a better starting point, and only a fine-tuning would be needed requiring a smaller amount of time. Also, the memory of GPUs is limited, and data transfers to and from the GPUs can become the bottleneck for self-training. The best solution to overcome these bottlenecks is to share the computational burden among multiple GPUs, which would increase the reconstruction speed and increase the possible size of the object to be reconstructed at the same time^37,38^.

## Conclusion

In this paper, we have proposed a physics-based deep image priors application on a limited-angle tomography problem without the need for training data. We have shown that by separating the reconstruction problem using the alternating direction method of multipliers framework, we can augment regularization for prior information in the deep image priors network. First, we have demonstrated that deep image priors can improve the reconstruction quality with a trade-off between the reconstruction accuracy and the reconstruction time. Then, we have proposed a cascaded architecture network to model the inverse tomography problem more accurately and demonstrated that performance could be further increased. Besides, we have shown that an improved reconstruction quality for data with high noise variance is possible using the proposed algorithm.

## Supplementary Information

Supplementary Information.

## Data Availability

The data and the code used in this study are available upon request.
