# Supplementary material for: Limited-angle computed tomography with deep image and physics priors
Source: Sci Rep. 2021 Sep 6;11:17740. doi: 10.1038/s41598-021-97226-2 (PMC8421356; doi:10.1038/s41598-021-97226-2)
Supplement: Supplementary file 1 — Supplementary Information. [file 41598_2021_97226_MOESM1_ESM.pdf]

Limited-Angle Computed Tomography with Deep Image and Physics Priors

Semih Barutcu[1,*], **Selin Aslan**[2], **Aggelos K. Katsaggelos**[1], **and Doğa Gürsoy**[1,2]

[1]Northwestern University, 2145 Sheridan Road, Evanston, IL, 60208, USA
[2]Argonne National Laboratory, 9700 South Cass Avenue, Lemont, IL, 60439, USA
[*]semihbarutcu@u.northwestern.edu

Supplementary Figures

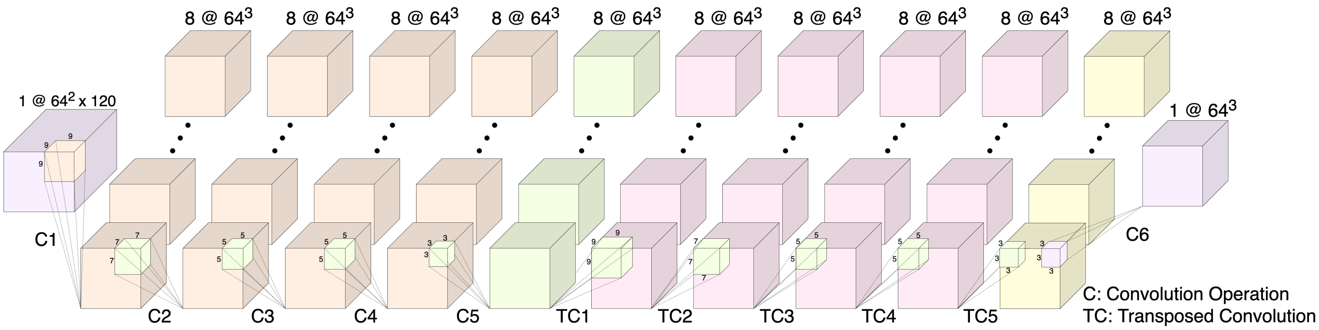

Figure S1. Network Architecture for Deep Image Priors Network

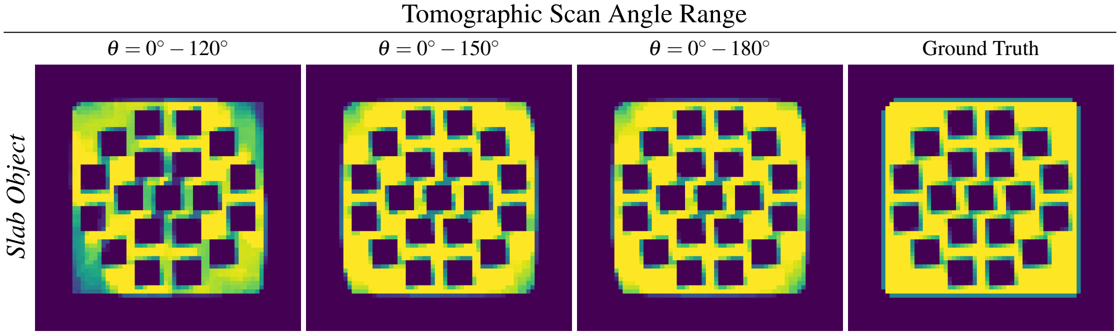

Figure S2. Reconstructions for 2D slab-shaped object[1] with square inclusions for different number of projections

References

1. Ching, D. J. & Gürsoy, D. XDesign: an open-source software package for designing x-ray imaging phantoms and experiments. https://github.com/tomography/xdesign.
